# A putative role for the aryl hydrocarbon receptor (*AHR*) gene in a patient with cyclical Cushing’s disease

**DOI:** 10.1186/s12902-020-0495-8

**Published:** 2020-01-29

**Authors:** Sunita M. C. De Sousa, Jim Manavis, Jinghua Feng, Paul Wang, Andreas W. Schreiber, Hamish S. Scott, David J. Torpy

**Affiliations:** 1 Endocrine and Metabolic Unit, Royal Adelaide Hospital, Adelaide, Australia; 2 Department of Genetics and Molecular Pathology, Centre for Cancer Biology, an SA Pathology and University of South Australia alliance, Adelaide, Australia; 3 School of Medicine, University of Adelaide, Adelaide, Australia; 4 ACRF Cancer Genomics Facility, Centre for Cancer Biology, an SA Pathology and University of South Australia alliance, Adelaide, Australia; 5 School of Pharmacy and Medical Sciences, University of South Australia, Adelaide, Australia; 6 School of Biological Sciences, University of Adelaide, Adelaide, Australia

**Keywords:** Cyclical Cushing’s, Aryl hydrocarbon receptor, Retinoid X receptor gamma, Whole exome sequencing, Clock genes

## Abstract

**Background:**

Apart from *PRKAR1A* mutations in a subset of cyclical Cushing’s syndrome due to primary pigmented nodular adrenocortical disease, the molecular basis of cyclical Cushing’s syndrome has not been investigated. We speculated that cyclical Cushing’s syndrome may be due to mutations in the clock genes that govern circadian rhythms, including the hypothalamic-pituitary-adrenal axis.

**Case presentation:**

A 47-year-old man presented with mass effects from a sellar lesion. He was ultimately diagnosed with cyclical Cushing’s disease due to a giant corticotrophinoma. We performed whole exome sequencing of germline and tumour DNA, SNP array of tumour DNA and tumour immunohistochemistry in order to detect variants in candidate circadian/pituitary-associated genes. We identified a rare germline missense variant in the aryl hydrocarbon receptor (*AHR*) gene, which has previously been indirectly linked to pituitary tumorigenesis and clock system disruption. The *AHR* variant was found in a highly conserved site involved in phosphorylation. It was predicted to be damaging by multiple in silico tools and AHR tumour immunohistochemistry demonstrated loss of the normal nuclear staining pattern, suggestive of an inactivating mutation. We also found a novel, damaging germline missense variant in the retinoid X receptor gamma (*RXRG*) gene, multiple somatic chromosomal gains (including *AHR*), and a somatic mutational signature consistent with oncogenesis that may have acted synergistically with the *AHR* variant.

**Conclusions:**

This is the first report of an *AHR* variant with predicted pathogenicity in the pituitary adenoma setting. Our preliminary data suggest that the highly conserved *AHR* gene may represent a link between pituitary tumorigenesis, the hypothalamic-pituitary-adrenal axis and the clock system. Further research may indicate a role for the gene in the development of cyclical Cushing’s disease.

## Background

Cyclical Cushing’s syndrome (CCS), characterised by intermittent biochemical hypercortisolism, accounts for approximately 20% of endogenous Cushing’s syndrome (CS) [1]. Cycles may last days to years, often with intraindividual consistency [1], suggesting an intrinsic fault in timekeeping. Competing theories for the pathogenesis of CCS include: episodic haemorrhage; periodic growth/death of tumour cells; persistence of negative feedback; and, in cyclical Cushing’s disease (CCD) only, altered hypothalamic control of the pituitary, via dopaminergic fluctuations for example [1].

The only known genetic cause of CCS is germline *PRKAR1A* mutations causing Carney’s complex, including the common manifestation of ACTH-independent CCS due to primary pigmented nodular adrenocortical disease (PPNAD) [2]. However, PPNAD-associated CS may be either cyclical or non-cyclical [2], and cyclicity is thus not necessarily explained by *PRKAR1A*. The molecular basis of CCS has not otherwise been investigated. We performed whole exome sequencing (WES) in a man with CCD to investigate the possibility that CCS may be due to perturbation in the clock genes responsible for circadian rhythms, including the hypothalamic-pituitary-adrenal (HPA) axis.

## Case presentation

A 47-year-old man was found to have optic disc swelling by his optometrist. MRI revealed a 7.1 cm sellar mass (Additional file 1: Figure S1), shown to be an ACTH-positive pituitary adenoma on transsphenoidal biopsy. He had a history of obesity, hypertension, gout and renal calculi but no cyclical symptoms or blood pressure fluctuations. Body mass index was 52.1 kg/m^2^ but he had no supraclavicular fat pads, Cushingoid striae, facial plethora, ecchymoses or proximal weakness. He had a right-sided oculomotor nerve palsy and right-sided proptosis and conjunctival injection suggesting ophthalmic vein compression. He was eupituitary apart from fluctuating ACTH-dependent cortisol production ranging from normal to 35-fold ULN (Additional file 1: Figure S1). He was diagnosed with CCD due to a giant corticotrophinoma with intermittent biochemical hypercortisolism, although the precise temporal cyclicity could not be defined prior to transcranial partial tumour resection 1 week later. Histopathology confirmed a corticotrophinoma with no significant mitotic activity and a Ki67 count of < 1%. He was eucortisolaemic immediately pre- and postoperatively with ACTH lowering from 376 ng/L (ULN 60) to 169 ng/L (Additional file 1: Figure S1). Postoperative complications included acute kidney injury, transient hyperglycaemia, pneumonia, deep vein thrombosis and central hypothyroidism. He later noticed improved BP control, reduced appetite and improved satiety with early but transient weight loss. Serial MRI showed a stable 4.2 cm tumour remnant (Additional file 1: Figure S1). Despite having typical CS comorbidities and postoperative complications, he has had no cyclical symptoms to guide the timing of investigations and no further episodes of overt hypercortisolism have been detected during intermittent testing. His family history is negative for endocrine tumours.

We performed WES of germline and tumour DNA and single nucleotide polymorphism (SNP) array of tumour DNA to identify sequencing variants and copy number variation in circadian/pituitary-associated genes. Tumour immunohistochemistry was performed to further evaluate the leading genetic variant of interest. Further details are provided in Additional file 1: Supplementary Methods.

## Results

**Fig. 1:**
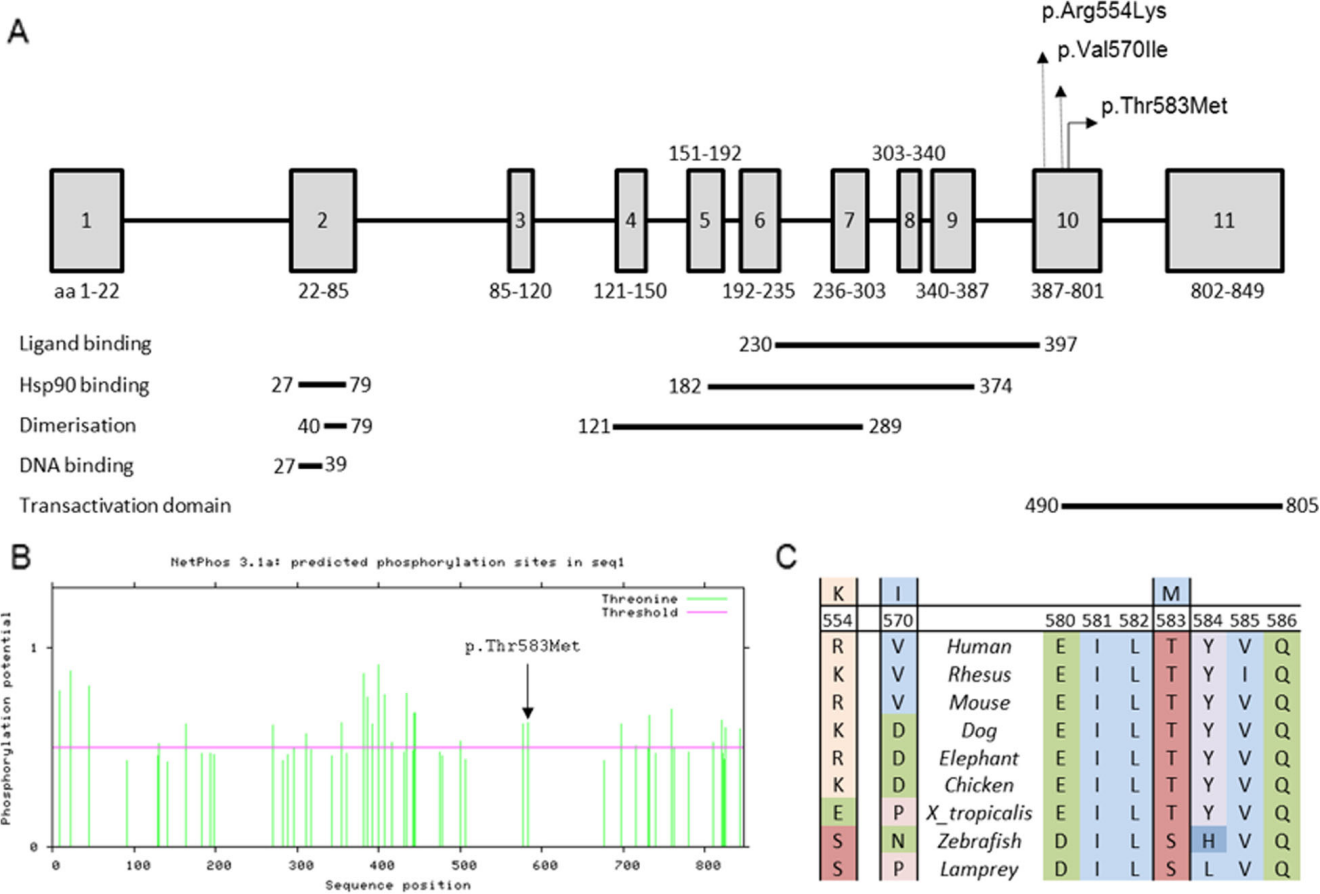
Position of aryl hydrocarbon receptor (*AHR*) variant. **a**. Schematic diagram of the *AHR* gene including the amino acid (aa) positions of all 11 exons (all coding). The location of the *AHR* variant in our patient (p.Thre583Met) is shown in exon 10, in addition to variants previously studied in the setting of acromegaly (p.Arg554Lys and p.Val570Ile). Functional domains based on extrapolations from mouse *Ahr* [4] are indicated. **b**. Threonine phosphorylation map of AHR by NetPhos 3.1 Server showing our patient’s variant located at a predicted phosphorylation site [3]. **c**. The alignment of protein sequences from different species showing our patient’s variant at aa position 583 to be more conserved compared to the previously studied variants at aa positions 554 and 570

**Fig. 2:**
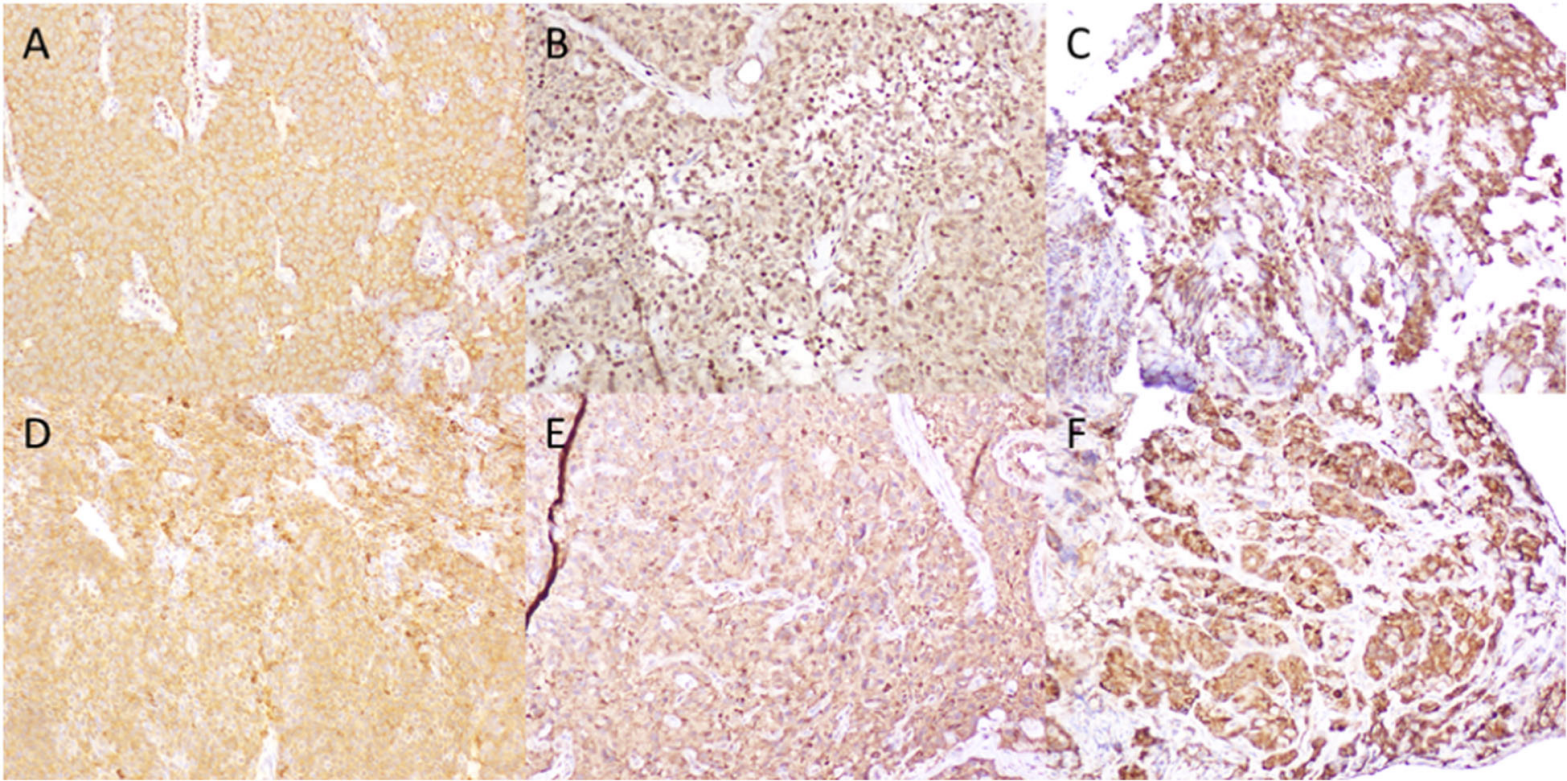
AHR and AIP tumour immunohistochemistry (100x magnification). **a**. Only cytoplasmic AHR staining was observed in the patient’s corticotrophinoma. **b**, **c**. Both cytoplasmic and nuclear AHR staining was observed in control corticotrophinoma specimens from two men with non-cyclical Cushing’s disease and no *AHR* variants. **d**-**f**. Cytoplasmic and membranous staining for the AHR chaperone, AIP, was found in the corticotrophinomas from the patient (**d**) and the two controls (**e**, **f**)

Germline DNA exhibited 14 rare, possibly damaging sequence variants in circadian/pituitary-associated genes (Additional file 1: Table S1). Tumour DNA did not exhibit any additional variants. Amongst the 14 germline variants of interest, only one variant was considered to be reliable and relevant to both circadian rhythm and pituitary tumorigenesis. This germline exon 10 *AHR* variant (GRCh37/hg19, Chr7:g.17379197C > T; ENST00000242057; c.1748C > T/p.Thr583Met) was present in the heterozygous state in both germline DNA (30/65 reads) and tumour DNA (86/177 reads). The variant is located in a site that is highly conserved and likely to be involved in phosphorylation [3] (Fig. 1). It was predicted to be damaging by five of six in silico tools. ExAC population allele prevalence is 0.01% with no homozygotes. It has been cited in Catalogue of Somatic Mutations in Cancer (COSMIC; cancer.sanger.ac.uk) in oesophageal adenocarcinoma [5]. Tumour immunohistochemistry showed restriction of AHR staining to the cytoplasm, whereas both cytoplasmic and nuclear AHR staining was seen in corticotrophinoma specimens from two male patients who had non-cyclical Cushing’s disease and no *AHR* variants on WES (Fig. 2). By contrast, staining for the AHR chaperone, AIP, showed cytoplasmic and membranous staining in all three corticotrophinomas.

**Fig. 3:**
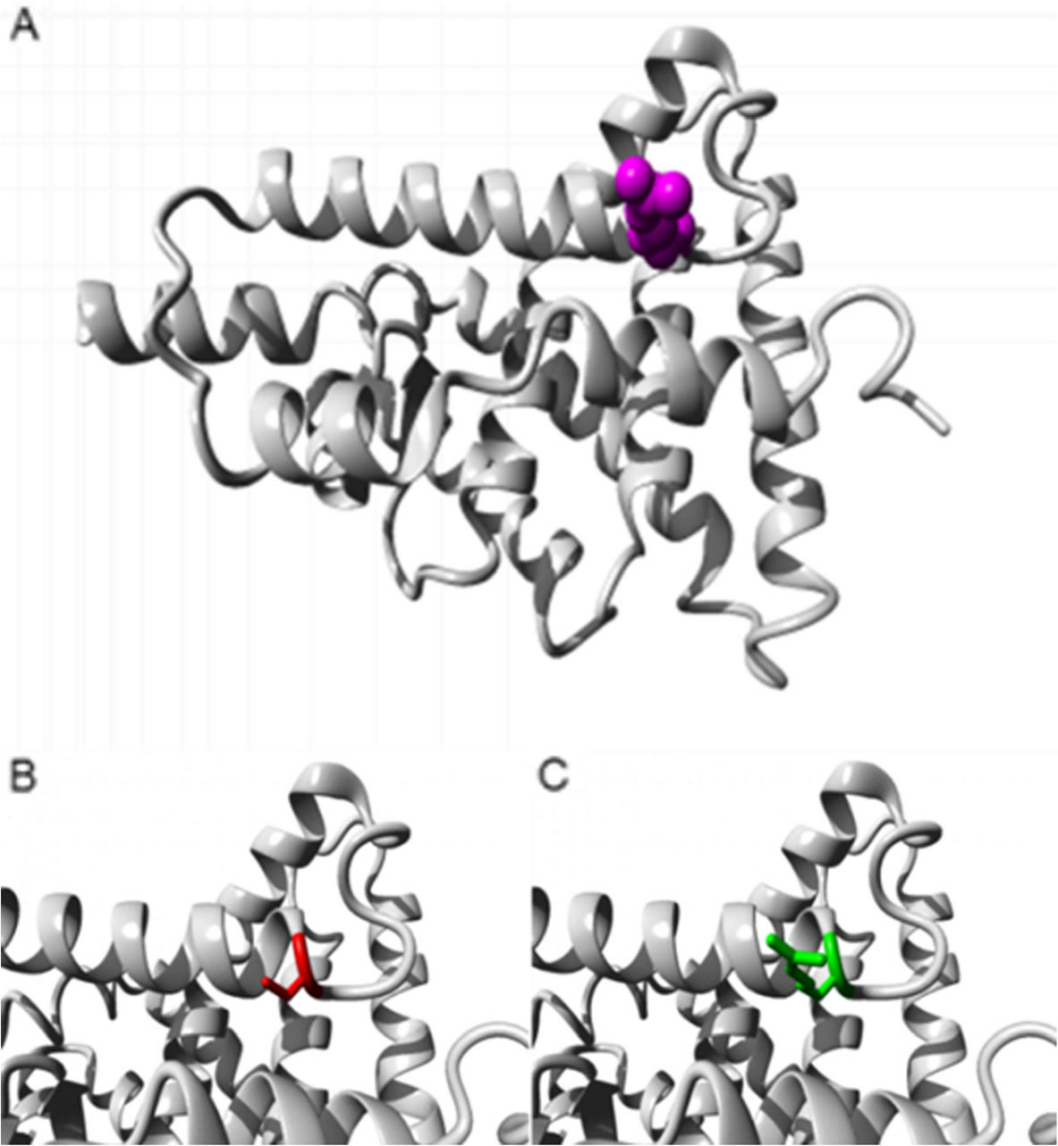
Structure of human wild-type and mutant retinoid X receptor gamma (RXRG) protein [6]. **a**. Arg286 (magenta) is part of the ligand binding domain and forms a salt bridge with glutamic acid at position 241 and glutamic acid at position 282. **b**-**c**. The substitution Arg286Cys (red) was shown through homology modelling to be smaller than the wild-type Arg286 (green), which may cause loss of external interactions. The charge of the wild-type residue is also lost by this substitution, which will disturb the ionic interaction made by the wild-type residue with nearby glutamic acid residues

WES also revealed a novel, damaging heterozygous germline *RXRG* variant (GRCh37/hg19, Chr1:g. 165379996C > T; ENST00000359842; c.856C > T/p.Arg286Cys) situated in the ligand binding domain (Fig. 3).

CNV analysis of tumour WES data revealed multiple chromosomal gains involving Chr 5,7,8,12-14,16,18–22 (Additional file 1: Figure S2). Orthogonal validation by SNP array showed arr(3,5,7)× 3,(8)× 4,(12,13,14)× 3,(16)× 4,(18,19,20,21,22)× 3. Involvement of Chr 7 by both WES and SNP array results indicated *AHR* copy number gain in the tumour, whilst the two different ploidy counts with Chr 8,16 tetrasomy and Chr 3,5,7,12-14,18–22 trisomy by SNP array suggested the possibility of multiple tumour clones. Tumour DNA disclosed 20 high confidence variants with a predominant mutational signature (Additional file 1: Figure S3) matching that seen in most cancer types [7].

## Discussion

The aryl hydrocarbon receptor (AHR) exists in a cytoplasmic complex with aryl hydrocarbon interacting protein (AIP), heat shock protein 90 and p23 protein [8]. Amongst many exogenous carcinogenic AHR ligands, the most potent is 2,3,7,8 tetrachlorodibenzo-*p*-dioxin (TCDD; dioxin) [9]. Activation by such ligands causes dissociation and nuclear translocation of AHR, followed by heterodimerisation with Ah receptor nuclear translocator (ARNT) and transcription of target genes involved in the cell cycle and clock system [8–11]. In the present study, we identified a germline Thr583Met *AHR* variant in a man with CCD. We speculate that this likely inactivating variant may have contributed to CCD development via a loss of tumour suppressor function in the pituitary and disruption of circadian/infradian rhythms.

To the best of our knowledge, *AHR* mutations have not been reported in patients with pituitary adenomas, including recent WES cohort studies of patients with Cushing’s disease [12, 13]. However, other lines of evidence support a tumour suppressor role for *AHR* in the pituitary. *AHR* is the major binding partner of *AIP*, which is an established pituitary tumorigenesis gene [10, 11]. Loss of AHR stabilisation is postulated to be contributory to *AIP*-associated pituitary tumorigenesis, with somatotrophinomas from patients with germline *AIP* mutations typically showing decreased cytoplasmic and absent nuclear AHR staining [11]. *AHR* may also have *AIP*-independent roles in pituitary tumorigenesis as *AHR* but not *AIP* expression is reduced in *GNAS*-mutated somatotrophinomas, and *AHR* activation increases the transcription of *CDKN1B*, which is another tumour suppressor gene implicated in pituitary and other endocrine tumours [10].

Whereas *AIP* germline mutations are most classically associated with somatotrophinomas, *AHR* may be more relevant to corticotrophinomas as in our patient. AHR immunostaining is found in the corticotroph-rich *pars intermedia* and normal corticotrophs demonstrate nuclear AHR immunostaining representative of activated *AHR* [11], although there have been no systematic AHR immunostaining studies of corticotrophinomas to date. Furthermore, pro-opiomelanocortin is overexpressed in mice and pituitary cell lines treated with the AHR ligand, dioxin [14].

The location of our patient’s *AHR* variant at a highly conserved phosphorylation site [3] supports the pathogenicity of this variant. Loss of the normal nuclear pattern of AHR tumour staining suggests that it is a loss-of-function variant causing failure of nuclear translocation. It is possible that the somatic Chr 7 trisomy amplified a dominant negative effect of this variant by increasing mutant dosage in the tumour. However, other evidence suggests that *AHR* may have proto-oncogenic effects. Recapitulating animal models, an excess of non-functioning pituitary adenomas (NFPA) and prolactinomas followed dioxin exposure from the 1976 Seveso accident in Italy [11], and acromegaly risk is 8-fold higher in Italian regions with high environmental exposure to AHR ligands such as cadmium [9]. The *AHR* SNPs, rs2066853 (c.1661G > A, p.Arg554Lys) and rs4986826 (c.1708G > A, p.Val570Ile), are over-represented in acromegalic patients in these regions with prevalences of 22.4 and 2.9%, respectively, compared to Caucasian ExAC allele prevalences of 9.9 and 0.3%, respectively [9]. Interestingly, these *AHR* SNPs and our patient’s variant all reside in exon 10, encoding the transactivation domain (Fig. 1) [6]. Exon 10 SNPs are associated with other neoplasms, including glioma, but pituitary studies have been limited to acromegaly [9]. The differential tumour suppressor and proto-oncogenic effects of *AHR* are yet to be fully elucidated but may depend on cell type.

*AHR* has an additional emerging role in the clock system, which entrains sleep, appetite, metabolism, locomotion and reproductive activity to 24 h day-night cycles [15, 16]. The upstream mediators of the clock system, circadian locomotor output cycle kaput (CLOCK) and brain-muscle-aryl hydrocarbon nuclear translocator-like protein 1 (BMAL1), heterodimerise and bind enhancer-box (E-box) regions in target genes, similarly to other members of the PER-ARNT-SIM (PAS) superfamily that includes AHR [15]. Separate to the canonical pathways of AHR/ARNT heterodimerisation and BMAL1/CLOCK heterodimerisation in the clock system, activated AHR can heterodimerise with BMAL1, indirectly affecting the regulation of diurnal patterns [16]. This is supported by greater amplitudes of downstream clock gene expression in Ahr-deficient versus wild-type mice [16]. On the other hand, pituitary adenomas are not reported in *Ahr*-deficient mice [16–18].

Other variants might have acted synergistically with the *AHR* variant, particularly the novel germline *RXRG* variant which has a Combined Annotation Dependent Depletion score of 34.0 (Additional file 1: Table S1). *RXRG* may act as a tumour suppressor gene in the pituitary as it is most highly expressed in the pituitary (GTEx; https://www.gtexportal.org/home/). Furthermore, *RXRG* belongs to the retinoid X nuclear receptor family that mediates the antiproliferative effects of retinoic acid, which has shown some efficacy in the treatment of Cushing’s disease [19]. Another novel, likely damaging *RXRG* variant (p.R317H) in the ligand binding domain segregated upon WES of a familial prolactinoma kindred [20], but *RXRG* has not previously been studied in Cushing’s disease. Our patient also had a somatic mutational signature typical of various cancer types [7]. Though only 20 high confidence variants were available for the signature analysis, this is not uncommon in pituitary tumours [21, 22] and the signature found raises the possibility of cooperation between the germline *AHR* and *RXRG* variants and somatic driver mutations.

A limitation of this case study is that the patient is currently in a prolonged state of normocortisolism, precluding additional investigations to demonstrate ongoing cyclicity. This is despite a significant tumour remnant, highlighting the discordance between structural and functional status in patients with CCD. Given the rarity of Cushing’s syndrome in general and CCD in particular, collaborative research is required to further examine the potential relationship between *AHR* and CCD raised by this case study.

## Conclusions

Preliminary data from this case study suggest that the highly conserved *AHR* gene may represent a link between pituitary tumorigenesis, the HPA axis and the clock system, implicating it in the development of CCD. With *AHR* known to be expressed in the pituitary, CCD may occur because of the combination of *AHR*-mediated pituitary tumorigenesis and disordered clock control of the HPA axis. Our patient’s somatic Chr 7 trisomy and germline *RXRG* variant might have been additive to his germline *AHR* variant, explaining how this variant can be seen in up to 1/10,000 individuals in population data despite the rarity of CCD. Alternatively, *AHR* variants might lead to cyclicity in individuals who happen to develop CS. Future research is required to determine whether *AHR* is a true pituitary tumorigenesis gene or a phenotypic modifier gene accounting for cyclicity in CS of various aetiologies.

## Supplementary information

**Additional file 1.** Supplementary Methods, Figures and Table.

## Data Availability

The datasets used and/or analysed during the current study are available from the corresponding author on reasonable request.

## Abbreviations

AHR: Aryl hydrocarbon receptor
AIP: Aryl hydrocarbon interacting protein
ARNT: Ah receptor nuclear translocator
BMAL1: Brain-muscle-aryl hydrocarbon nuclear translocator-like protein 1
CCD: Cyclical Cushing’s disease
CCS: Cyclical Cushing’s syndrome
CLOCK: Circadian locomotor output cycle kaput
COSMIC: Catalogue of Somatic Mutations in Cancer
CS: Cushing’s syndrome
E-box: Enhancer-box
GTEx: Genotype-Tissue Expression
HPA: Hypothalamic-pituitary-adrenal
NFPA: Non-functioning pituitary adenoma
PAS: PER-ARNT-SIM
PPNAD: Primary pigmented nodular adrenocortical disease
RXRG: Retinoid X receptor gamma
SNP: Single nucleotide polymorphism
TCDD: 2,3,7,8 tetrachlorodibenzo-p-dioxin
ULN: Upper limit of normal
WES: Whole exome sequencing
