# Supplementary material for: A putative role for the aryl hydrocarbon receptor (AHR) gene in a patient with cyclical Cushing’s disease
Source: BMC Endocr Disord. 2020 Jan 29;20:18. doi: 10.1186/s12902-020-0495-8 (PMC6988286; doi:10.1186/s12902-020-0495-8)

*BMC Endocrine Disorders* Appendix:

**A putative role for the aryl hydrocarbon receptor (*AHR*) gene in a patient with cyclical Cushing’s disease**

Sunita MC De Sousa, Jim Manavis, Jinghua Feng, Paul Wang, Andreas W Schreiber, Hamish S Scott, David J Torpy

**Supplementary Methods**

Tissue specimens from the corticotrophinoma were obtained at the time of surgery and stored in RNALater at -80°C. A fresh blood sample was obtained for extraction of germline DNA from peripheral blood leucocytes. Both tumour and germline DNA were extracted using commercially-available kits (Qiagen) according to the manufacturer’s protocols. Whole exome sequencing (WES) of tumour DNA and paired germline DNA was performed using Roche NimbleGen SeqCap EZ MedExome Target Enrichment Kit with mean depth of coverage of 100X and 95% of bases covered ≥20X. Bioinformatic analysis was performed at the ACRF Cancer Genomics Facility in Centre for Cancer Biology SA Pathology (Adelaide, Australia) using GATK HaplotypeCaller (1) to detect small variants (typically <50bp) and in-house scripts to analyse copy number variation (CNV).

Raw WES data were filtered for variants that were: rare (<1% population); possibly damaging (by snpEFF impact, splicing/binding predictions, GERP or CADD); and of high quality (Phred-scaled score >50 and depth of coverage >30X). All heterozygous and homozygous variants were then manually filtered for associations with the clock system, circadian rhythm, pituitary tumorigenesis or corticotroph function. Raw data were also compared against a list of candidate genes based on existing literature: *USP8*, *CABLES1*, *AIP*, *MEN1*, *CDKN1B*, *SDHA*, *SDHB*, *SDHC*, *SDHD*, *PRKAR1A*, *CDH23*, *NR3C1*, *EGFR*, *POMC*, *SMARCA4*, *HDAC2*, *GNAS*, *DICER1*, *CLOCK*, *PER1*, *PER2*, *PER3*, *CRY1*, *CRY2*, *TIMELESS*, *ARNTL*, *TIPIN*, *TBX19*, *HSP90AA1*, *NR2C2*, *AVPR1B*, *GPR101*, *PROP1*, *NPAS2*, *BHLHE40*, *BHLHE41*, *NFIL3*, *DBP*, *HLF*, *NR1D1*, *NR1D2*, *RORA*, *RORB*, *RORC*, *GSK3B*, *BTRC*, *FBXL3*, *FBXL21*, *FBXL15*, *RXRG* and *TH*. Both germline and tumour variants were considered. Whilst the slight overrepresentation of ectopic ACTH syndrome in cyclical versus non-cyclical Cushing’s syndrome (2) suggests that the putative molecular cause is tumour-specific and therefore a somatic mutation, the ubiquity of cyclicity amongst the various Cushing’s syndrome aetiologies suggests that individuals have a germline genetic predisposition that produces cyclicity if Cushing’s syndrome ever develops.

High confidence somatic variants were also used to generate a tumour mutational signature for comparison against the Sanger Institute’s Signatures of Mutational Processes in Human Cancer (https://cancer.sanger.ac.uk/cosmic/signatures) (3).

High density SNP array of tumour DNA was performed using the Illumina Infinium CytoSNP-850K BeadChip to validate CNV results from WES data analysis.

For immunohistochemistry the following antibodies were used: a rabbit polyclonal antibody raised against human AHR (Novus Biologicals, CO, USA, Cat # NBP1-89975) and a mouse monoclonal antibody raised against human AIP/ARA9 (clone 35-2, Novus Biologicals, CO, USA, Code # NB100-127) using a standard streptavidin-biotinylated immunoperoxidase technique. In brief, sections were dewaxed using xylene and then rehydrated through alcohols. Sections were then rinsed twice in PBS (pH 7.4) for 5min. Antigen retrieval was then performed using Citrate buffer (pH 6). Slides were allowed to cool and washed twice in PBS (pH 7.4), then endogenous peroxidase activity was quenched. Non-specific proteins were blocked using normal horse serum for 30min. The polyclonal AHR antibody was applied at a dilution of 1:100 and the monoclonal AIP antibody at a dilution of 1:1600 at room temperature overnight. The following day, the sections were given two washes in PBS, then either a biotinylated anti-rabbit secondary (Vector Laboratories, USA, Cat # BA-1000) or a biotinylated anti-mouse secondary (Vector Laboratories, USA, Cat # BA-2000) was applied for 60min at room temperature. Following two PBS washes, the slides were incubated for 1hr at room temperature with a streptavidin-conjugated peroxidase tertiary (Pierce, USA, Cat # 21127). Sections were then visualised using diaminobenzidinetetrahydrochloride (DAB), washed, counterstained with haematoxylin, dehydrated, cleared and mounted on glass slides.

**References**

1. Van der Auwera GA, Carneiro MO, Hartl C, et al. From FastQ data to high confidence variant calls: the Genome Analysis Toolkit best practices pipeline. Curr Protoc Bioinformatics. 2013;43:11.0.1-33.

2. Meinardi JR, Wolffenbuttel BH, Dullaart RP. Cyclic Cushing's syndrome: a clinical challenge. Eur J Endocrinol. 2007;157(3):245-54.

3. Alexandrov LB, Nik-Zainal S, Wedge DC, et al. Signatures of mutational processes in human cancer. Nature. 2013;500(7463):415-21.

**Supplementary Table 1 Germline variants in genes of interest due to relationship with the clock system and/or pituitary gland**

| Gene symbol | Reason for interest | Evidence against pathogenicity | Chr position | AA change | ExAC % | CADD | GERP | Tumour DNA count* | Germline DNA count* |
| --- | --- | --- | --- | --- | --- | --- | --- | --- | --- |
| *AHR* | Inhibits *PER1* by repressing CLOCK-ARNTL/BMAL1-mediated transcriptional activation of PER1 (uniprotkb); binds known pituitary tumorigenesis gene, *AIP* |  | 7:17379197 | p.Thr583Met, c.1748C>T | 0.01 | 24.9 | 5.28 | 91,86 | 35,30 |
| *ASIC3* | Strongly expressed in pituitary (uniprotkb) | Benign by 3 of 4 in silico tools | 7:150747594 | p.Arg238Gly, c.712C>G | 0.042 | 25.1 | <2 | 72,82 | 33,34 |
| *CDH23* | Known pituitary tumorigenesis gene | Synonymous | 10:73377084 | p.Ala361Ala, c.1083C>T | 0.014 | 16.66 | 4.56 | 78,83 | 45,35 |
| *CDH23* | Known pituitary tumorigenesis gene | 0.3% pop frequency | 10:73492073 | p.Arg1354Cys, c.4060C>T | 0.259 | 31 | 4.02 | 73,67 | 41,28 |
| *CLOCK* | Canonical clock gene | 0.5% pop frequency; low mutant load in germline, mutant absent in tumour | 4:56304529 | p.Gln760del, c.2278_2280delCAG | 0.479 | <10 | 4.47 | 36,0 | 46,5 |
| *ERBB4* | Strongly expressed in pituitary (uniprotkb) | Intronic; low mutant load in germline, mutant absent in tumour | 2:212543728 | c.1622+49G>T |  | <10 | 2.52 | 188,0 | 42,7 |
| *GALR2* | Neuromodulator in hypothalamic-pituitary axis (refseq_gene_summary) | 0.8% pop frequency | 17:74073386 | p.Ser346Arg, c.1038C>G | 0.831 | 23.2 | 2.72 | 58,40 | 35,30 |
| *PHLPP1* | Involved in circadian control by regulating the consolidation of circadian periodicity after resetting (uniprotkb function) | Intronic | 18:60582301 | c.2804+60T>C |  | <10 | 2.8 | 24,33 | 21,13 |
| *PRLHR* | Encodes prolactin releasing hormone receptor; only expressed in pituitary and pituitary adenomas (uniprotkb) | Synonymous | 10:120354258 | p.Leu167Leu, c.499C>T |  | <10 | 4.48 | 95,82 | 37,50 |
| *PTTG1IP* | Binds pituitary transcription factor, securin encoded by *PTTG1* (uniprotkb & refseq_gene_summary) | Low mutant load in germline, mutant absent in tumour | 21:46276193 | p.Cys121del, c.361_363delTGC | 0.196 | <10 | 4.85 | 37,0 | 53,6 |
| *RPTOR* | Strongly expressed in pituitary (uniprotkb) | Synonymous | 17:78921066 | p.Phe1060Phe, c.3180C>T | 0.2 | 15.07 | 5.25 | 107,105 | 54,45 |
| *RXRG* | Suspected pituitary tumorigenesis gene |  | 1:165379996 | p.Arg286Cys, c.856C>T |  | 34 | 5.1 | 37,52 | 19,21 |
| *SIX3* | Acts with HESX1 to control cell proliferation via Wnt/beta-catenin pathway in pituitary development (uniprotkb) | 0.5% pop frequency; low mutant load in germline, mutant absent in tumour | 2:45169429 | p.Gly69del, c.205_207delGGC | 0.517 | <10 | 2.94 | 40,0 | 36,4 |
| *THRB* | Encodes thyroid hormone receptor | 0.5% pop frequency | 3:24206606 | p.Ala80Ala, c.240C>G | 0.487 | 15.46 | 5.78 | 64,30 | 31,20 |

*Abbreviations: AA, amino acid; CADD, Combined Annotation Dependent Depletion score; Chr, chromosome; ExAC, Exome Aggregation Consortium database; GERP, Genomic Evolutionary Rate Profiling score; HESX1, homeobox expressed in ES cells 1; pop, population; * DNA reads of wild-type allele, mutant allele.*

**Supplementary Fig. 1 Radiological and biochemical features of the patient**

A-D. MRI showing corticotrophinoma at diagnosis and 1, 5 and 9 months following partial tumour resection by transcranial approach. At baseline, the 7.1cm sellar mass invaded the sphenoid and bilateral cavernous sinuses and skull base, and impinged upon the third ventricle, right temporal lobe and midbrain. E. Graphical representation of cyclical cortisol production relative to upper limit of normal for each parameter. *Abbreviations: 24UFC, 24-hour urinary free cortisol; ACTH, adrenocorticotrophic hormone; serum cort, random serum cortisol; xULN, calculated by absolute result divided by upper limit of normal.*


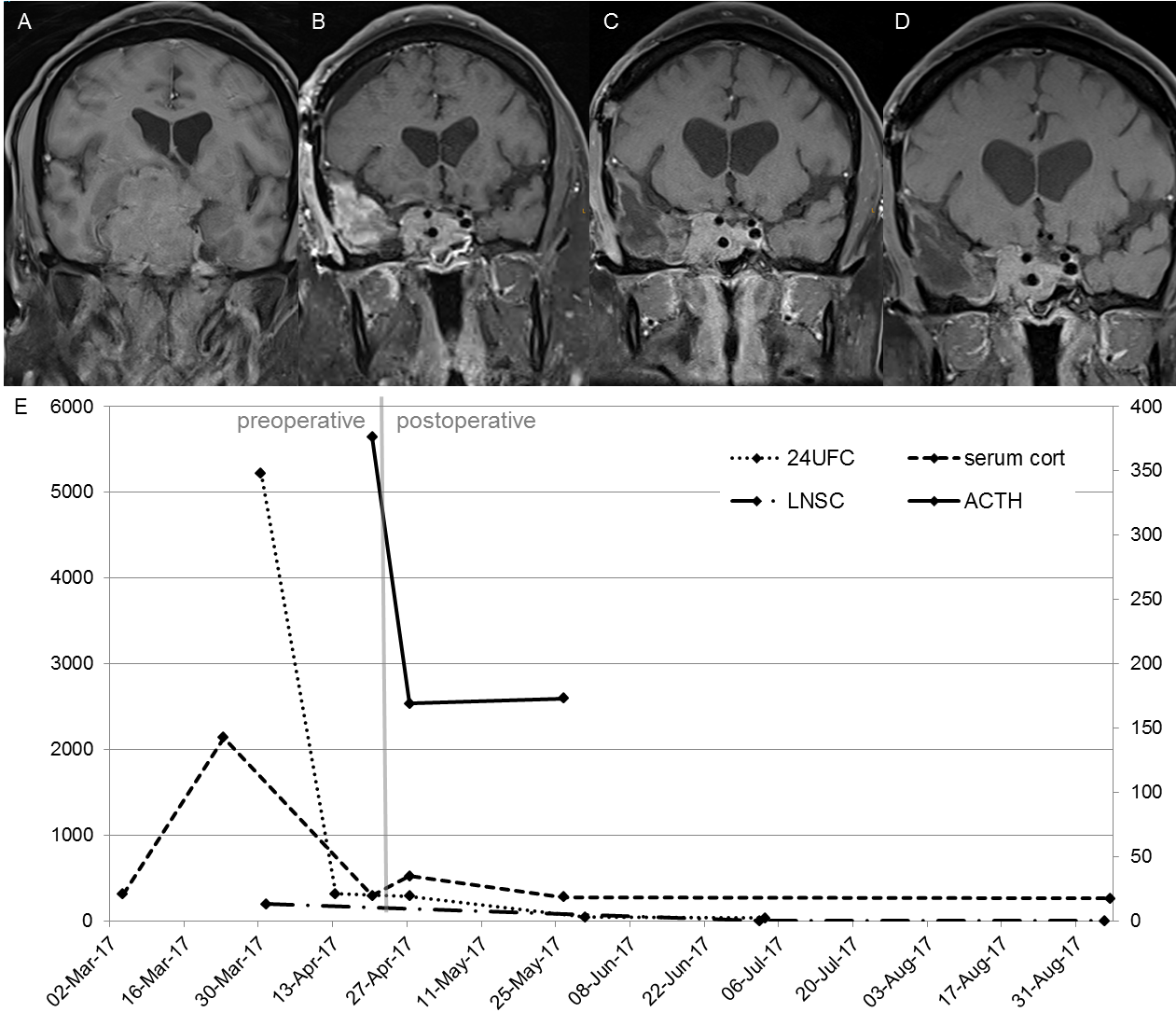


E


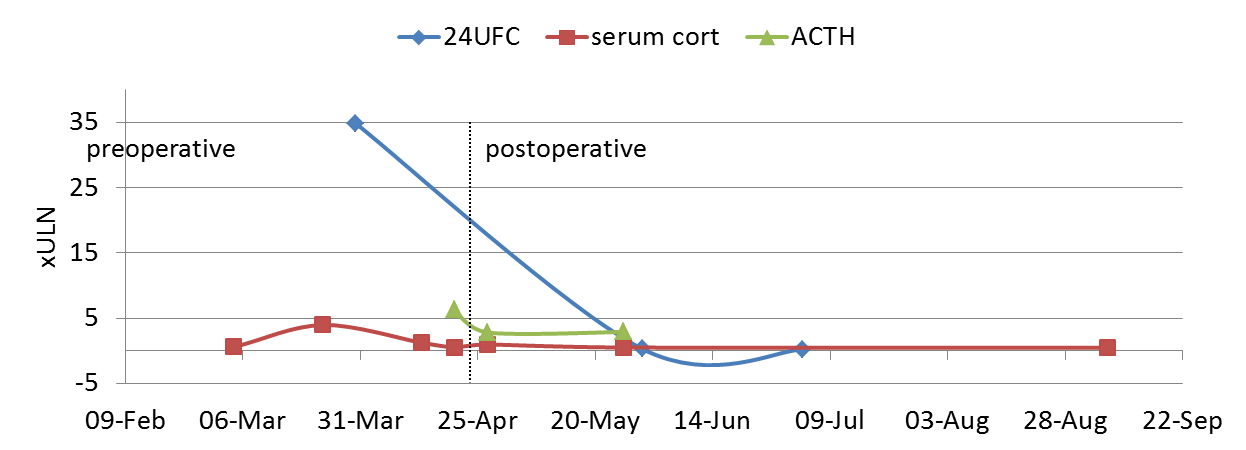


**Supplementary Fig. 2 Tumour ploidy count**

Ploidy plots derived from whole exome sequencing copy number variation analysis of tumour DNA, showing maximal gains in Chr 8,16 and intermediate gains in Chr 5,7,12-14,18-22. SNP array confirmed Chr 8,16 tetrasomy and Chr 5,7,12-14,18-22 as well as Chr 3 trisomy.


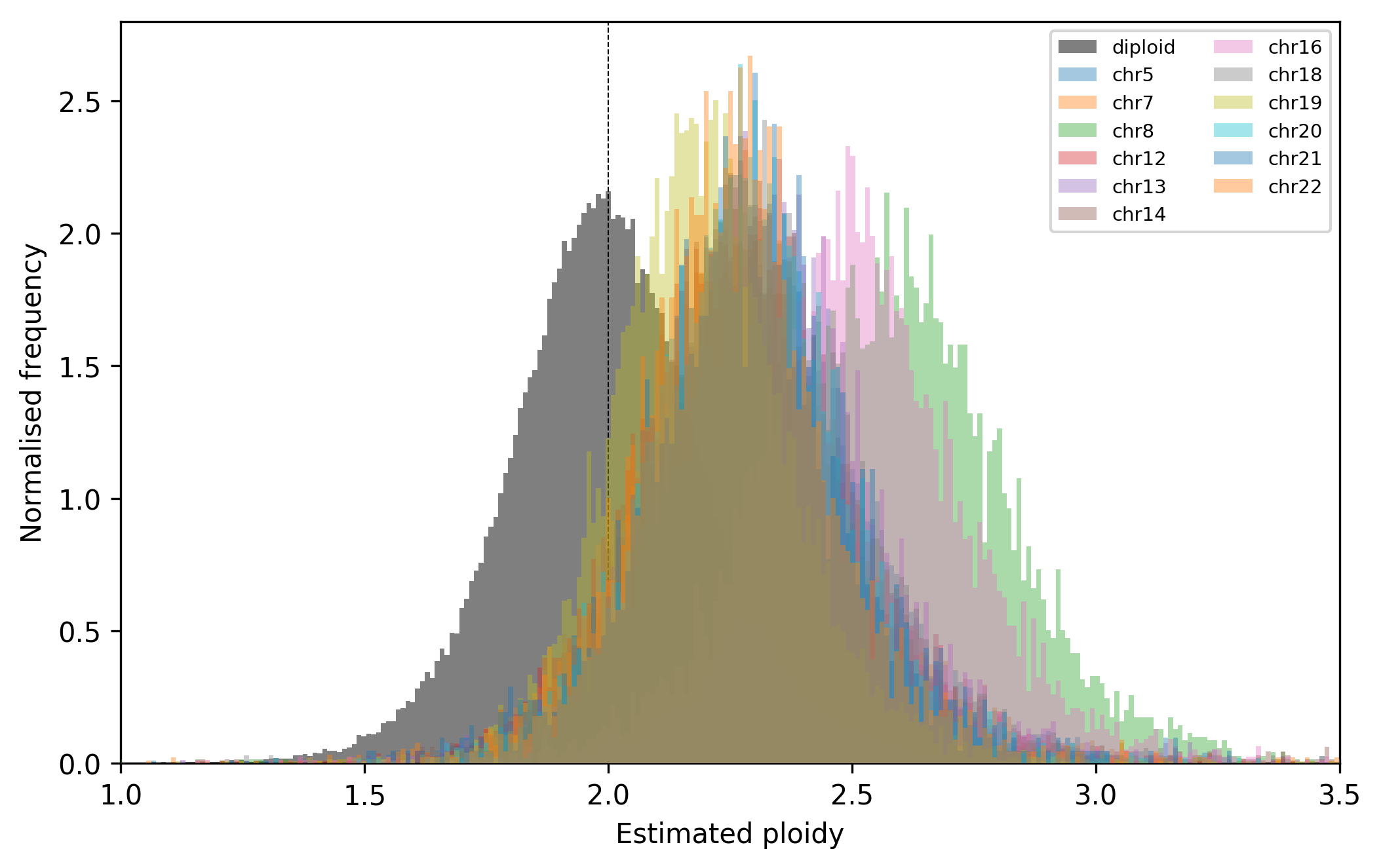


**Supplementary Fig. 3 Mutational signature of tumour DNA**

Plot of high confidence somatic variants according to Sanger Institute Mutational Signature types 1-30 showing the closest match to Signature 5 which has been found in all cancer types and most cancer samples though the aetiology of this signature is unknown (https://cancer.sanger.ac.uk/cosmic/signatures) (3).


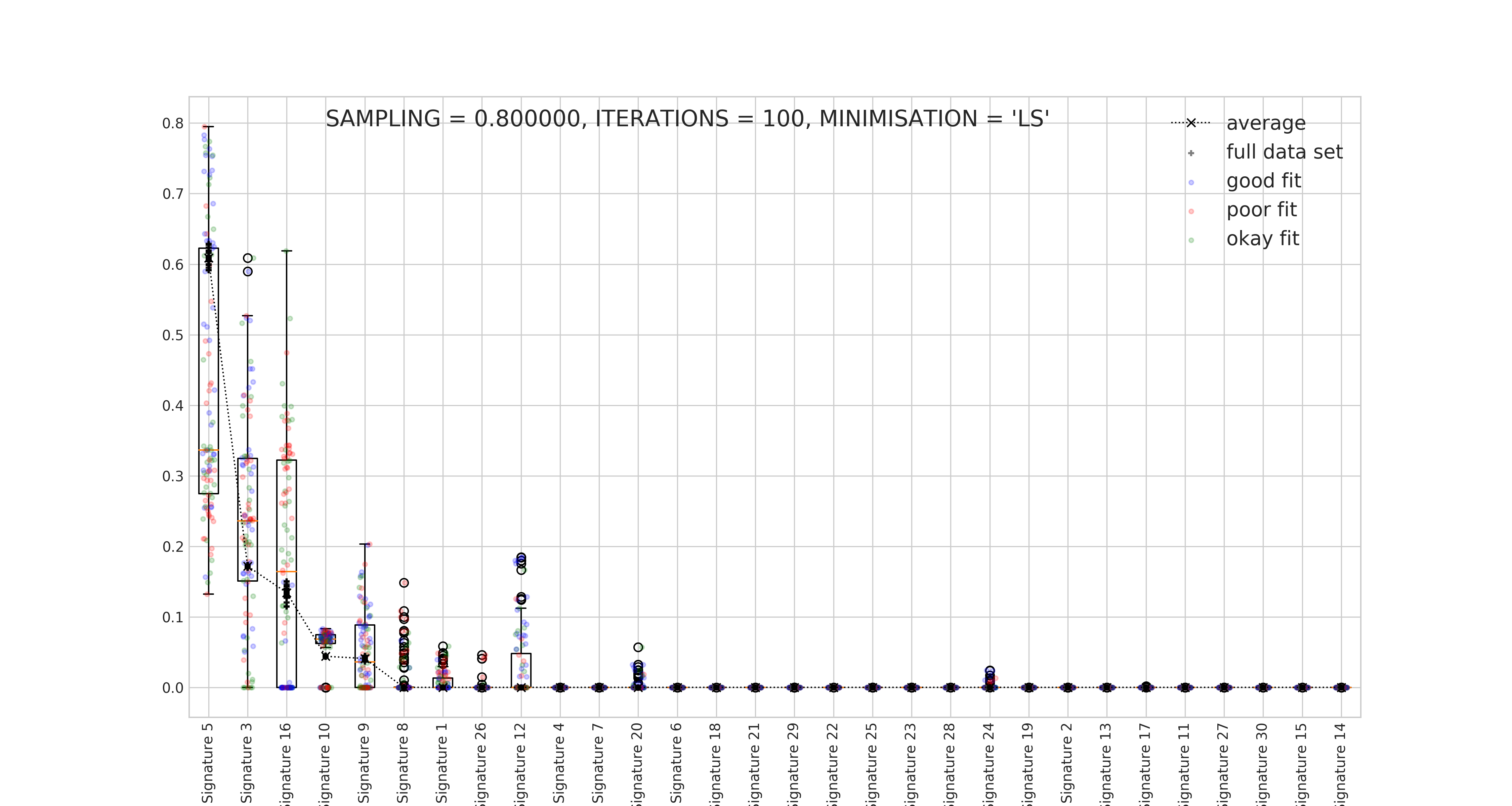

Supplement: Supplementary file 1 — Additional file 1. Supplementary Methods, Figures and Table. [file 12902_2020_495_MOESM1_ESM.docx]
